# Monitored Anesthesia Care with Dexmedetomidine Supplemented by Midazolam/Fentanyl versus Midazolam/Fentanyl Alone in Patients Undergoing Pleuroscopy: Effect on Oxygenation and Respiratory Function

**DOI:** 10.3390/jcm10163510

**Published:** 2021-08-09

**Authors:** Andreas Kostroglou, Emmanouil I. Kapetanakis, Paraskevi Matsota, Periklis Tomos, Konstantinos Kostopanagiotou, Ioannis Tomos, Charalampos Siristatidis, Michail Papapanou, Tatiana Sidiropoulou

**Affiliations:** 1 Second Department of Anesthesiology, Attikon University Hospital, National and Kapodistrian University of Athens, Rimini 1, 12462 Athens, Greece; andreaskostr@gmail.com (A.K.); matsota@yahoo.gr (P.M.); 2 Department of Thoracic Surgery, Attikon University Hospital, National and Kapodistrian University of Athens, Rimini 1, 12462 Athens, Greece; emmanouil_kapetanakis@yahoo.com (E.I.K.); periklistomos@hotmail.com (P.T.); kostop@hotmail.co.uk (K.K.); 3 Second Department of Pulmonology, Attikon University Hospital, National and Kapodistrian University of Athens, Rimini 1, 12462 Athens, Greece; etomos@hotmail.com; 4 Assisted Reproduction Unit, Second Department of Obstetrics and Gynecology, Aretaieion Hospital, Medical School, National and Kapodistrian University of Athens, Vas. Sofias 76, 11528 Athens, Greece; csyristat@med.uoa.gr; 5 Second Department of Obstetrics and Gynecology, Aretaieion Hospital, Medical School, National and Kapodistrian University of Athens, Vas. Sofias 76, 11528 Athens, Greece; mixalhspap13@gmail.com

**Keywords:** pleuroscopy, thoracoscopy, monitored anesthesia care, dexmedetomidine, midazolam

## Abstract

Although pleuroscopy is considered a safe and well tolerated procedure with a low complication rate, it requires the administration of procedural sedation and analgesia. The purpose of this study was to assess the effects of dexmedetomidine administration on oxygenation and respiratory function in patients undergoing diagnostic or therapeutic pleuroscopy. Through a prospective, single center, cohort study, we studied 55 patients receiving either a dexmedetomidine intravenous infusion supplemented by midazolam/fentanyl (Group DEX + MZ/F) or a conventional sedation protocol with midazolam/fentanyl (Group MZ/F). Our primary outcome was the changes in lung gas exchange (PaO_2_/FiO_2_ ratio) obtained at baseline and at predetermined end points, while changes in respiratory mechanics (FEV1, FVC and the ratio FEV1/FVC) and PaCO_2_ levels, drug consumption, time to recover from sedation and adverse events were our secondary endpoints (NCT03597828). We found a lower postoperative decrease in FEV1 volumes in Group DEX + MZ/F compared to Group MZ/F (*p* = 0.039), while FVC, FEV1/FVC and gas exchange values did not differ between groups. We also found a significant reduction in midazolam (*p* < 0.001) and fentanyl consumption (*p* < 0.001), along with a more rapid recovery of alertness postprocedure in Group DEX + MZ/F compared to Group MZ/F (*p* = 0.003), while pain scores during the postoperative period, favored the Group DEX + MZ/F (*p* = 0.020). In conclusion, the use of intravenous dexmedetomidine during pleuroscopy is associated with a smaller decrease in FEV1, reduction of the consumption of supplementary sedatives and analgesics and quicker awakening of patients postoperatively, when compared to midazolam/fentanyl. Therefore, dexmedetomidine administration may provide clinically significant benefits in terms of lung mechanics and faster recovery of patients undergoing pleuroscopy.

## 1. Introduction

Pleuroscopy is being increasingly used by chest physicians and has become, after bronchoscopy, the second most commonly utilized endoscopic procedure in the field of respiratory medicine [1,2]. It is considered to be an important part of a specialist pleural disease service [3]. Although it is considered as a safe procedure with very low complication rates in experienced hands [4,5], it requires the administration of procedural sedation and analgesia. The latter is essential for both alleviating patients’ discomfort by reducing their anxiety and minimizing the pain, and for providing better procedural conditions for the operator [6]. Therefore, the presence of an anesthesiologist during the procedure is mandatory, especially for the management of high-risk patients [7].

Dexmedetomidine is a potent, highly selective α2-adrenoreceptor agonist, endowed with a unique sedation pattern, which mimics normal sleep [8]. Its onset time is less than five minutes, while its maximum effect is expected to occur within the first 15 min [9]. In specific, the patient is sedated but readily rousable, and able to cooperate throughout the procedure [10,11]. In addition, it is easily titrated to the desired level of sedation [12], exhibiting minimal respiratory depression within therapeutic concentrations (in contrast to midazolam or propofol) [8], and has proved to be a safe and very promising agent in a wide variety of procedures [13,14], with minimal side effects [15]. The benefits of dexmedetomidine have been well documented in bronchoscopy patients [16,17] and in video-assisted thoracoscopic surgery (VATS) patients [18,19].

Furthermore, dexmedetomidine is an attractive sedative agent with a unique feature of maintaining the patient’s respiratory function and providing a cooperative sedation during pleuroscopy. Notably, clinical trials on its efficacy and safety during the procedure are currently lacking. The purpose of this prospective, single center trial was to assess the effects of dexmedetomidine administration on oxygenation and respiratory function in patients undergoing diagnostic or therapeutic pleuroscopy for a pleural effusion compared to conventional monitored anesthesia care (MAC) technique with a midazolam/fentanyl combination.

## 2. Materials and Methods

This prospective, cohort study was conducted at the Department of Anesthesiology, Pulmonology and Thoracic Surgery, in University Hospital “Attikon”, Athens, Greece, from 5 August 2018 to 30 June 2020. The trial received ethical approval by the Hospital’s Scientific and Bioethics Committee (decision number: 2376/31-10-2017) and was registered prior to commencement at Clinical Trials.gov (registration number: NCT03597828).

### 2.1. Patient Population/Eligibility Criteria

Inclusion criteria included: patients scheduled to undergo pleuroscopy for a pleural effusion; aged 18 years old or over; American Society of Anesthesiologists class I–IV. All patients provided written informed consent prior to their enrollment in the study. 

Exclusion criteria included: any use of general anesthesia within 7 days prior to study enrollment; α2-agonist or antagonist intake within 14 days prior to the scheduled pleuroscopy procedure; use of intravenous (IV) opioid within 1 h, or oral or intramuscular opioid within 4 h from the initiation of the study drug administration; a New York Heart Association class >3; acute unstable angina; an acute myocardial infarction confirmed by laboratory findings in the past 6 weeks; bradycardia at rest (≤50 bpm); systolic blood pressure ≤90 mm Hg; a 2nd or/and 3rd degree atrioventricular block, unless the patient had a pacemaker in situ; severe functional hepatic or renal disease; morbid obesity (body mass index ≥40 kg/m^2^); severe restrictive interstitial lung disease.

### 2.2. Groups, Anesthesia Management and Preoperative Assessment

Baseline spirometric and arterial blood gas analysis (ABG) parameters were obtained in all patients the day before surgery. Patients were assessed for eligibility and non-randomly allocated prior to their procedure on a 1:1 basis either to a continuous dexmedetomidine infusion supplemented by midazolam/fentanyl (Group DEX + MZ/F) or to a midazolam/fentanyl only sedation (Group MZ/F). Standard monitoring (electrocardiogram, SpO_2_, invasive blood pressure measurement, end tidal carbon dioxide measurement), and supplemental oxygen via an air-entrainment mask at an FiO_2_ of 50% was utilized on all patients prior to initiation of the procedure. The initial loading dose of dexmedetomidine was 1 μg/kg administered over 10 min, followed by a 0.5 μg/kg/hr continuous infusion. The group MZ/F received a saline infusion to blind anesthesiologists and surgeons from group allocation. Both infusions were prepared by a nurse who was aware of group allocation but was not involved further in the procedure and data collection. Ten minutes after starting the study drug administration, patients were assessed for their level of sedation using the Observer’s Assessment of Alertness/Sedation scale (OAA/S), and any patient having a score ≥3 received IV midazolam in 0.5 mg doses, repeated until the OAA/S was ≤2. The OAA/S scale was developed to measure the level of alertness in subjects who are sedated and is well documented and verified [20]. Briefly, each number corresponds to a level of responsiveness where 5: Awake and responds to name, spoken in normal tone, 4: Lethargic response to name, spoken in normal tone, 3: Responds only after name called loudly and/or repeatedly, 2: Responds only after name called loudly and after mild shaking of body and 1: No response after name is called loudly with mild shaking.

### 2.3. Procedure, Intraoperative and Postoperative Assessments

All subjects received local anesthesia at the thoracoport insertion point with lidocaine 1.5% and ropivacaine 0.75% according to patient’s weight (lidocaine <3 mg/kg and ropivacaine <2 mg/kg). Inadequate sedation during pleuroscopy was counteracted with IV midazolam boluses of 0.5 mg, repeated as needed to achieve an OAA/S score of ≤2. Pain during the procedure was treated with IV fentanyl at 25 μg boluses repeated as necessary, if verbally expressed by the patient, or if the anesthesiologist determined the presence of pain when verbal communication was not possible (transient tachycardia or hypertension, or detection of patient movement during surgical stimulus). The infusion of dexmedetomidine or saline was discontinued after wound closure.

Intraoperatively, repeat ABG analysis was performed 30 min after the start and at the end of the procedure. OAA/S scores and standard vital signs were obtained every 5 min throughout the procedure. Adverse effects of fentanyl, such as muscle rigidity, episodes of desaturation and hypopnea/hypoxemia, were registered. The time to reach an OAA/S score of 5 from the end of sedation was recorded. Further data recorded postoperatively included the cumulative dose of dexmedetomidine, midazolam and fentanyl consumption, the volume of crystalloids infused, adverse events (hypoxemia, bradycardia, hypotension etc.) and pain score measured with an 11-point numerical rating scale (NRS) (0 = no pain, 10 = worst pain). In NRS scores >3, we administered 1 g of acetaminophen intravenously. Blood gas analysis was obtained every 30 min until patient’s discharge from the Postoperative Anesthetic Care Unit (PACU). Patient’s and operator’s satisfaction scores were also registered (4-point scale—unsatisfied/satisfied/good/excellent). Postoperative lung function spirometric values were assessed approximately 18 h after surgery, administered by the same study investigator, blinded to the group allocation, using the same device (Spirobank II; MIR; Rome, Italy) in order to minimize interobserver variability.

### 2.4. Sample Size Calculation, Primary and Secondary Outcomes

A sample size estimation was performed at the design stage of the study. Lung oxygenation expressed by PaO_2_/FiO_2_ ratio during lung ventilation, comprised our primary outcome. To extrapolate the expected effect size, PaO_2_/FiO_2_ ratio mean ± SD values from a previously reported similar study were utilized [19]. Data from that study demonstrated an expected effect size (Cohen’s d = 0.933) corresponding to a large (>79%) magnitude difference. Based on this difference, it was calculated that a sample size of 26 patients was required in each group for a power of 90%, with a two-sided significance level (alfa value) of 0.05. Patients were assigned on an alternating 1:1 ratio to avoid selection bias to either group. Secondary outcomes measured included changes in Forced Expiratory Volume at 1 s (FEV1), Forced Vital Capacity (FVC), FEV1/FVC, partial pressure of oxygen (PaO_2_) to fraction of inspired oxygen (FiO_2_) ratio (PaO_2_/FiO_2_), and partial pressure of carbon dioxide (PaCO_2_) before and after the procedure. Secondary outcomes included time to reach OAA/S scale = 5 after the procedure, cumulative drug consumption, patient and surgeon satisfaction rate, and incidence of adverse events.

### 2.5. Statistical Analysis

Normality was tested using the Kolmogorov–Smirnov test. To calculate the effect of dexmedetomidine on respiratory function the percentage change (Δ%) for the values of FEV1, FVC, FEV1/FVC, PaO_2_/FiO_2_ ratio and PaCO_2_ was calculated for each patient using the formula: [ΔV% = (V1 − V2)/V1 × 100], where V1 is the measured value at baseline and V2 represents the measured value at 24 h after the procedure for FEV1, FVC, or FEV1/FVC, and at discharge from the PACU for PaO_2_/FiO_2_ ratio and PaCO_2_. Subsequently, a comparison of average percentage change values was performed. Time to OAA/S score of 5, after the discontinuation of the infusion, was analyzed by Kaplan–Meier survival analysis, with a comparison between groups using the log-rank test. Comparisons between the two patient groups was performed using the Student’s *t*-test or the Mann–Whitney U test for continuous variables and the χ^2^ test for categorical variables. Multiple comparisons were performed with either one-way ANOVA or the Kruskal-Wallis tests, as appropriate. Continuous data are reported as mean ± standard deviation (SD) or as mean [95% CI]; categorical data are reported as numbers (percentages). A p-value of 0.05 or less was considered statistically significant. Statistical analysis was carried out with the SPSS v23.0 software (SPSS Inc., Chicago, IL, USA).

## 3. Results

**Figure 1:**
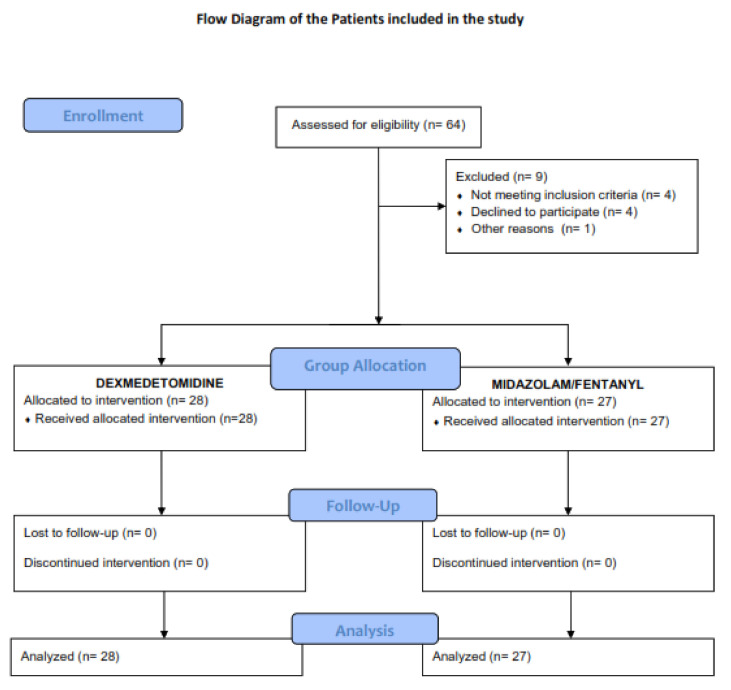
Flow Diagram of Study Participants.

From 5 August, 2018 to 30 June, 2020, 64 patients scheduled for pleuroscopy were assessed for eligibility, and four patients who did not meet the inclusion criteria were excluded from the study. Four more patients refused enrollment in the study, while another had a general anesthetic before the study for surgical reasons. Therefore, a total of 55 patients were enrolled and assigned into the two study groups (Figure 1).

The baseline demographic and procedural characteristics of the patients in both groups were similar, with no clinically meaningful differences (Table 1), although talc pleurodesis was performed more frequently in the DEX + MZ/F group (92% vs. 70%, *p* = 0.037). There were no episodes of muscle rigidity, hypoxemia or desaturation registered intraoperatively. 

**Figure 2:**
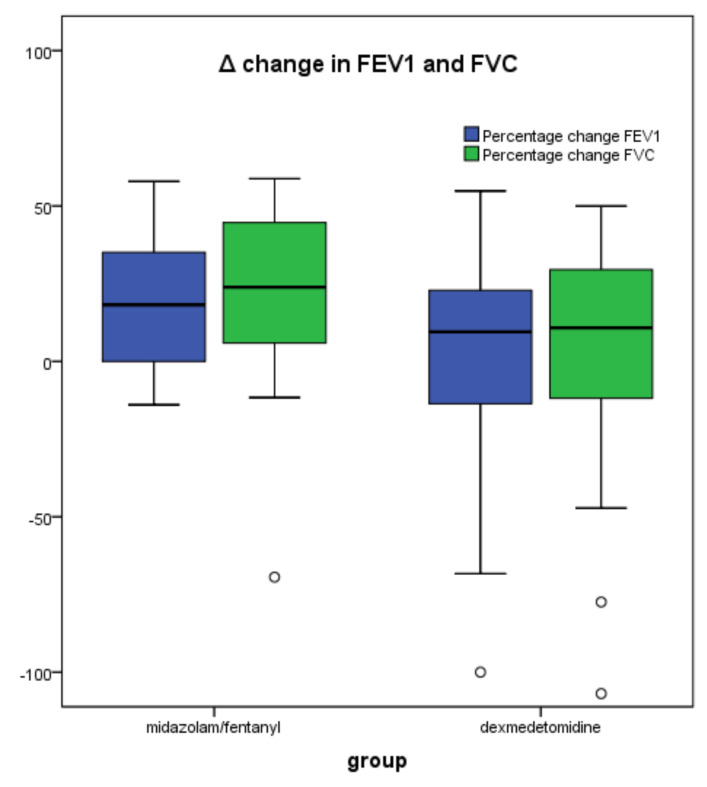
Percentage change in FEV1 and FVC before and after the procedure. The addition of dexmedetomidine to midazolam/fentanyl is associated with less decrease in FEV1 postoperatively. Bars represent means; boxes represent interquartile ranges; error bars represent ranges and dots represent outliers. Abbreviations: FEV1, forced expiratory volume in 1 min; FVC, forced vital capacity.

Assessment of lung spirometric values and gas exchange are reported in Table 2. Preoperative values of FEV1 differed between the two groups, being lower in the DΕΧ + ΜΖ/F group. Nonetheless, FEV1 values decreased less after the procedure in the DEX + MZ/F group (2 (-13–16) vs. 20 (11–29), *p* = 0.039) (Table 2, Figure 2). FVC volumes also showed a higher decrease in the MZ/F group without reaching statistical significance (22 (10–33) vs. 4 (-11–18), *p* = 0.054]. PaCO_2_ levels demonstrated a higher increase during the procedure in the MZ/F group, but after the end of the procedure they were similar to the DEX + MZ/F group values (Table 2). There were no other differences reported between the two groups.

Midazolam (1.4 ± 1 mg vs. 4 ± 1.3 mg, *p* < 0.001) and fentanyl (87 ± 45 μg vs. 218 ± 77 μg, *p* < 0.001) consumption were reduced in the DEX + MZ/F group compared to group MZ/F (Table 1). Furthermore, NRS pain scores were lower in the DEX + MZ/F group (mean (95%CI): 1 (0.33–1.67)) compared to the MZ/F group (mean (95%CI): 2.54 (1.5–3.6), *p* = 0.02). Patient satisfaction was higher with the use of dexmedetomidine (Table 1). There was no incidence of postoperative nausea and vomiting in the two groups, and no other complications such as hypoxia, bradycardia or hypotension were reported.

**Figure 3:**
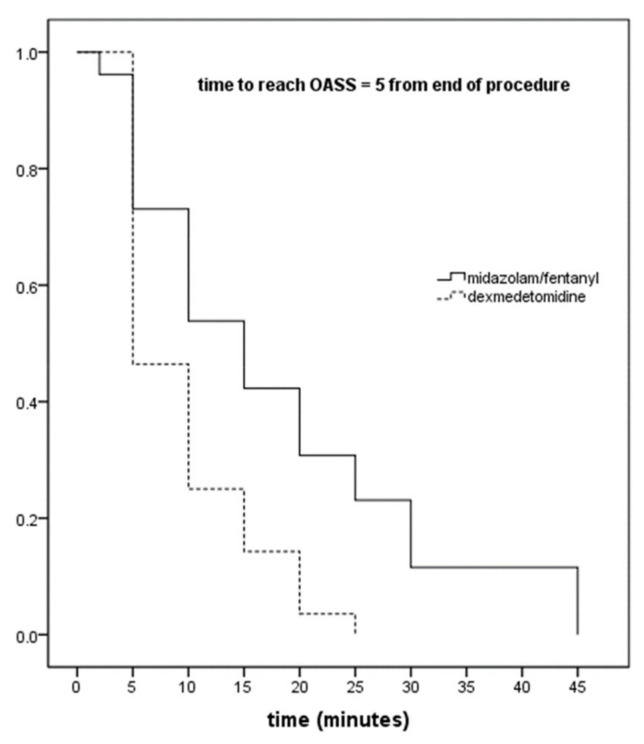
Kaplan–Meier analysis for time to reach an OAA/S = 5 between the two groups. Log-rank test. Abbreviation: OAA/S, Observer’s Assessment of Alertness/Sedation scale.

Kaplan–Meier analysis for the time to reach an OAA/S score of 5 (maximum) is depicted in Figure 3. Log-rank test revealed a significant difference between the two groups (mean (95% CI), MZ/F: 17.8 min (12.7–22.8), DEX + MZ/F: 9.5 min [(7.2–11), *p* = 0.003).

## 4. Discussion

The purpose of this single center, prospective study was to assess the effects of dexmedetomidine on the perioperative oxygenation and respiratory mechanics in patients undergoing pleuroscopy. Lung oxygenation by means of the PaO_2_/FiO_2_ ratio and ventilation (PaCO_2_ levels) were similar between the two groups. A slight increase in PaCO_2_ levels in the MZ/F group was observed intraoperatively, probably due to the increased opioid consumption in that group. We detected a lower postoperative decrease in FEV1 volumes in the dexmedetomidine group, although preoperative values were higher in the MZ + F group. Forced vital capacity and their index (FEV1/FVC) were comparable between groups. Concerning the other secondary endpoints preset for this study, we observed a significant reduction in midazolam and fentanyl consumption in this group. Consequently, a more rapid recovery of alertness after the procedure was observed in the same group. Moreover, during the postoperative period, patients who received dexmedetomidine also reported lower pain scores.

During pleuroscopy, the application of medically induced pneumothorax in the open, non-dependent hemithorax causes lung volume loss, mediastinal shift and paradoxical respiration, all of whom, in combination with sedation, may result in clinically significant hypoventilation and hypercarbia [21]. These processes may also impede venous return from the inferior vena cava reducing the cardiac preload, resulting in notable hemodynamic effects, similar to those observed during thoracotomy [22]. Overall, the final effect could be significant respiratory compromise, denoting that procedural sedation and analgesia during pleuroscopy is not a risk-free procedure [23].

Although the application of dexmedetomidine in pleuroscopy is restricted to isolated case reports, albeit in high-risk patients [24,25], a clear benefit of its use has been shown in patients undergoing VATS in well conducted studies [18,19]. In specific, Lee and colleagues [18] reported an increase in the quality of recovery and in FEV1 using dexmedetomidine, after VATS, similarly to our study. They advocate that the analgesic effect of dexmedetomidine both direct, on the α-adrenergic receptor, and indirect, through the potentiation of opioids given intraoperatively and prolongation of their analgesic effects, might contribute to an improved postoperative pulmonary function, a result also confirmed by another study [26]. Indeed, pain after thoracic surgery is known to influence postoperative pulmonary function [27,28]. Similar to the abovementioned studies, it is possible that this pain-modulating effect might have also contributed to the observed beneficial effect of dexmedetomidine infusion on FEV1 values in our study. The same authors revealed a higher PaO_2_/FIO_2_ ratio, significantly lower dead space ventilation and a higher dynamic compliance with the use of dexmedetomidine in patients scheduled for VATS with moderate chronic obstructive pulmonary disease [19]. In morbidly obese patients with restrictive disease and scheduled for bariatric surgery, dexmedetomidine infusion resulted in a higher PaO_2_/FiO_2_ ratio, higher compliance, decreased dead space and plateau pressure [29]. Conversely to these studies, we failed to detect any difference in PaO_2_/FiO_2_ ratio with the use of dexmedetomidine.

Dexmedetomidine was also found to ameliorate postoperative cognitive dysfunction in elderly patients undergoing VATS procedures [30]. In our study, we did not aim to detect postoperative cognitive disturbances, nevertheless, we detected a reduction in midazolam and fentanyl consumption, which consequently led to a faster recovery of alertness, as suggested by the high OAA/S scores. Indeed, patients receiving dexmedetomidine recovered approximately 10 min earlier than the control group. This observation might have a marginal clinical significance in this study in which the procedure duration was short, but could be of utmost importance in longer procedures and in other settings. Elderly patients or those with chronic renal disease might markedly benefit from the use of dexmedetomidine as the pharmacokinetics of the drug are not influenced by renal clearance, in contrast to midazolam or fentanyl [8]. This finding has also been confirmed in a recent meta-analysis in which the prophylactic use of the drug, when compared with a placebo, was related to a decline in the incidence of postoperative delirium [31].

The present study, similarly to previous studies, demonstrated an analgesic effect of dexmedetomidine and higher patient and operator satisfaction scores [11,32,33]. The drug can modulate nociceptive transmission in the central nervous system by acting on both supraspinal and spinal sites. Activation of subtype α-2 adrenoceptors in the dorsal horn of the spinal cord inhibits release of neurotransmitters, preventing propagation of neural activity in nociceptive pathways [34]. Interestingly, in our study, patients receiving dexmedetomidine reported higher satisfaction scores of the overall procedure, even though talc pleurodesis, one of the most recognized causes of severe pain in pleuroscopy [35], was significantly more frequent in this group. This observation could be explained by its higher analgesic effect compared to the conventional protocols, as well as the shorter period to regain alertness experienced in this group. 

Although we have carefully and adequately conducted a prospective study ending up with favorable outcomes of the examined drug, there are limitations. Firstly, as in every single center study, the results could differ in other centers based on population and physician diversities and preferences. Secondly, although the superior outcome on FEV1 observed in this study is novel regarding pleuroscopy patients, there are various reports highlighting the effects of dexmedetomidine on lung function, albeit in other settings [16,18,19]. Thirdly, the study was focused on respiratory parameters and secondary effects in cognition were not examined. Nevertheless, the analgesic effect, as well as the protective effect, of dexmedetomidine on cognitive function demonstrated in our study has been extensively reported in other studies and in different settings, and our results can be considered confirmatory regarding these outcomes. Finally, there are further limitations linked to the nature of the study rendering it less powerful, mainly due to the lack of randomization used to allocate patients in the two groups, inserting bias.

To the best of the authors’ knowledge, this is the first clinical study evaluating the role of dexmedetomidine in the context of pleuroscopy. Further, properly conducted trials are warranted to adequately address these outcomes specifically and to prove or disprove these findings.

## 5. Conclusions

To date, this is the first clinical study using dexmedetomidine with conventional midazolam/fentanyl sedation in pleuroscopy procedures. Our study demonstrated that the use of dexmedetomidine does not improve PaO_2_/FiO_2_ ratio during pleuroscopy. It provides, however, a smaller decrease in FEV1 after pleuroscopy, significantly reduces the consumption of other sedatives and analgesics, and accelerates the awakening of patients after the procedure. Therefore, dexmedetomidine administration may provide clinically significant benefits in terms of lung mechanics and faster recovery of patients undergoing pleuroscopy. 

## Figures and Tables

**Table 1:** Demographic and Procedural Clinical Data.

	Group DEX + MZ/F (*n* = 28)	Group MZ/F (*n* = 27)	*p-*Value
Age (years)	72 ± 11	67 ± 8	0.66
BMI (kg/m^2^)	26 ± 5	28 ± 6	0.88
Sex (male/female)	18/10	17/10	0.835
ASA status (II/III/IV)	5/22/1	11/16/0	0.105
Smoking, *n* (%)	14 (50%)	14 (52%)	0.493
Procedure duration (minutes)	53 ± 14	55 ± 14	0.568
Drainage amount (ml)	967 ± 481	1024 ± 386	0.629
Talc pleurodesis	26 (92%)	19 (70%)	0.037
Dexmedetomidine (μg/kg)	1.52 ± 0.41	-	-
Crystalloids (ml)	439 ± 303	469 ± 298	0.716
Fentanyl (μg/kg)	1.21 ± 0.65	2.82 ± 1.17	<0.001
Midazolam (mg/kg)	0.02 ± 0.014	0.05 ± 0.02	<0.001
NRS (0–10 point)	1 (0.33–1.67)	2.54 (1.5–3.6)	0.02
Acetaminophen i.v. (y/n)	6/22	18/9	<0.001
PONV	0	0	-
Patient Satisfaction (unsatisfied/satisfied/good/excellent)	0/3/4/21	0/0/10/17	0.045
Surgeon Satisfaction (unsatisfied/satisfied/good/excellent)	1/1/5/20	0/1/3/24	0.697

DEX, dexmedetomidine; MZ/F, midazolam/fentanyl; BMI, body mass index; ASA, American Society of Anesthesiologists; n, number; NRS, numerical rating scale pain score; PONV, postoperative nausea and vomiting. Data are reported as means ± SD, mean (95% CI of mean) or numbers (percentages).

**Table 2:** Respiratory and Clinical Outcomes.

	Group DEX + MZ/F (*n* = 28)	Group MZ/F (*n* = 27)	*p-*Value
**FEV_1_ predicted (%)**			
Preoperative	54 (46–62)	68 (59–78)	0.021
Postoperative	48 (43–54)	51 (46–57)	0.496
ΔFEV1 predicted	2 (−13–16)	20 (11–29)	0.039
**FVC predicted (%)**			
Preoperative	54 (47–61)	71 (62–80)	0.005
Postoperative	48 (43–54)	51 (46–56)	0.495
ΔFVC%	4 (−11–18)	22 (10–33)	0.054
**FEV_1_/FVC (%)**			
Preoperative	80 (76–84)	80 (75–85)	0.960
Postoperative	78 (75–81)	80 (78–83)	0.174
ΔFEV1/FVC	1 (−4–6)	-2 (-9–5)	0.419
**PaO_2_/FiO_2_ ratio**			
Preoperative	301 (269–333)	367 (343–390)	0.001
− Intraoperative			
− 30′	299 (241–357)	278 (236–321)	0.564
− End	299 (228–370)	257 (221–294)	0.275
Postoperative − 30′ − Discharge	297 (251–343) 306 (258–353)	296 (256–337) 347 (295–399)	0.987 0.235
ΔPaO_2_/FiO_2_ratio (%)	−10 (31–11)	6 (-6–18)	0.178
**SpO_2_ (%)**			
Preoperative	97.6 (96.4–98.8)	99 (98.4–99.5)	0.172
− Intraoperative			
− 30′	97.4 (96–98.7)	97.3 (95.9–98.8)	0.894
− End	97.4 (96.1–98.6)	97.8 (96.7–98.8)	0.910
Postoperative − 30′ − Discharge	99 (98.5–99.5) 99 (98.4–99.7)	97 (96–98.3) 98.4 (97.7–99.1)	0.002 0.035
**PaCO_2_ (mmHg)**			
Preoperative	39 (36–41)	37 (35–39)	0.213
− Intraoperative	47 (44–50) 44 (40–47)	52 (49–56) 51 (47–54)	0.02 0.005
− 30′			
− End			
Postoperative − 30′ − Discharge	43 (41–46) 43 (41–46)	45 (43–46) 43 (41–45)	0.419 0.715
ΔPaCO_2_ (%)	−14 (−19–(−8))	−19 (−25–(−12))	0.227

FEV1, forced expiratory volume in 1 min; Δ, difference; FVC: forced vital capacity, PaO_2_: partial pressure of oxygen, FiO_2_, fraction of inspired oxygen; PaCO_2_: partial pressure of carbon dioxide. Data are reported as mean (95% CI).

## Data Availability

The data presented in this study are available on request from the corresponding author.
